# Paradoxical Personality and Academic Achievement in College Students From Buenos Aires

**DOI:** 10.5964/ejop.v11i4.898

**Published:** 2015-11-27

**Authors:** Agustín Freiberg Hoffmann, María Mercedes Fernández Liporace

**Affiliations:** a National Council of Scientific and Technical Research, University of Buenos Aires, Buenos Aires, Argentina; University College Cork, Cork, Ireland

**Keywords:** paradoxical personality, college students, academic achievement, creativity

## Abstract

This paper presents a study on paradoxical personality, defined as a distinctive feature in creative persons, developed with 350 college students from Buenos Aires. Goals aimed at describing and analysing possible significant differences of paradoxical traits in students from diverse majors representing seven different fields of study, and examining the relationship between each bipolar trait and academic achievement. The sample was composed of 7 groups (n = 50 by group) representing fields of study typically offered in public universities, Biology, Computer Science, Engineering, Law, Nutrition, Psychology, and History of Art. Analyses by career provided descriptive information about students of these majors, concerning their paradoxical personality profiles. Correlational studies verified significant associations between academic achievement and most paradoxical traits in majors such as Computer Science, Nutrition and Psychology. Results are discussed regarding practical outcomes and teaching programs.

## Introduction

During 1999 UNESCO promoted means aimed at expanding and ensuring access to universities, as well as at improving learning quality (UNESCO, 2009a). World-wide statistics estimate an annual average increase of 4.6% in applications between 1970 and 2009, indicating that college students’ admission doubles every 15 years (UNESCO, 2009b). The Argentinean system of higher education (public and private) observes a similar trend. Data show an increment of freshmen, represented by an average annual growth rate of 1.7% from 2000 to 2010 (Ministerio de Educación, Secretaría de Políticas Universitarias, 2011).

Despite this rise in admissions, high dropout rates and a decrease in academic achievement are repeatedly reported. From the total number of freshmen in every field of study at public universities, only 22.68% corresponds to new students, whereas the remaining 77.32% is due to changes from one major to another (Ministerio de Educación, Secretaría de Políticas Universitarias, 2010). In fact, 58% of freshmen drops out or decides a change from one major to another (Pintos, 2012). Most institutions inform that only one in four freshmen achieves a college degree (“Decanos y rectores,” 2012). Official statistics show Natural and Formal Sciences as the most affected by dropout, with a graduation percentage of 5.58%, followed by Applied Sciences with 13.91%, Human Sciences with 16.88%, Medicine and related careers with 18.67%, and Social Sciences with 27.71% (Ministerio de Educación, Secretaría de Políticas Universitarias, 2010). It is openly admitted that the equity of accessing college must be improved but the pertinence and quality of contents must be addressed as well. Such actions would have an impact on students’ well-being, increasing graduation rates, and training more competitive and competent professionals.

In view of the above, analysing variables potentially related to academic achievement (taken as the official indicator of learning, beyond every reasonable debate) and to students permanence in the system arises as a matter of interest. Particularly, international organisations (e.g. CEPAL, 2011; UNESCO, 1998), as well as recent research (e.g. Hammershøj, 2014; Karwowski, Gralewski, Lebuda, & Wiśniewska, 2007; Likeschová & Tichá, 2013; Salakhatdinova & Palei, 2015; Santamaría & Sánchez, 2012; Sawyer, 2006; Wu & Albanese, 2013) highlight the role of creative skills in the academic milieu, stressing the importance of research on the concept, facing the design of teaching programs to enhance learning processes. The relevance of creativity in higher education stands out considering future outcomes in professional life once a degree is obtained.

### The Role of Creativity in (Higher) Education

Diverse national and international organisations point out creativity as a major and desirable skill in college students. For instance, UNESCO states that creative analysis must be trained by higher education (UNESCO, 1998). Thus, when obtaining a degree, former students would be proficient in learning self-management, as well as in trying new solutions for actual professional problems. In addition, new technologies demand the set in motion of policies oriented to stimulate creativity in students, in order to enhance the use of these skills as learning resources (CEPAL, 2011). Two decades ago, both CEPAL and UNESCO coincided in asserting that knowledge and technologies are winning wider spaces in class. Its gradual expansion produced academic transformations, demanding the development of new abilities in students (CEPAL-UNESCO, 1991). Moreover, the importance of creativity has been locally recognised by the Argentine Law of Higher Education #24521, which declares that individual differences, teamwork, and personal and group creativity must be preserved and encouraged (Ministerio de Educación, Secretaría de Políticas Universitarias, 2011). Different studies tabled the potential benefits of creativity in class, verifying its positive relationship with academic achievement (e.g., Getzels & Jackson, 1962; Ghayas & Malik, 2013; Naderi, Abdullah, Aizan, Sharir, & Kumar, 2010; Pérez Fabello & Campos, 2007). Some others found that different types of creativity are associated to different professional competences (Cheung, Rudowicz, Yue, & Kwan, 2003; Esquivias Serrano & De la Torre, 2010; Kelly & Daughtry, 2008). Besides, creative ability has been held as facilitating learning and the ability to relate informational items apparently unconnected (Martínez Zaragoza, 2010). Creative processes are associated to intrinsic motivation, to higher levels of involvement in academic activities, and to the use of strategies to relate new contents to previous knowledge (Miller & Dumford, 2014).

Examining creativity entails multiple approaches, such as describing the process, the person, the products, and the environment. This study is set to the analysis of creativity through the personal dimension, particularly by means of personality traits (Garbanzo Vargas, 2007; Tejedor Tejedor & García Valcárcel, 2007). Research on personality provides evidences about the individual way of perceiving and processing information, since it emphasises personal feelings, thoughts, and actions (Bridges, 1926; Sutin, Costa, Evans, & Zonderman, 2013). Focusing on students arises as a matter of interest due to the fact that academic achievement is influenced by the interaction between personality traits and teaching methods (Crozier, 2001). Following these ideas, analysing personality traits linked to creative skills in college students aims at the comprehension of their relationships with academic achievement and, ultimately, with learning. That would allow the design of specific teaching programs and special guidance in every particular group or case.

## The Creative Person

Csikszentmihalyi (1996) describes the creative person as capable to get the modification of the field of his/her expertise using thoughts and actions. According to this assertion, the creative dimension can be analysed through multiple psychological dimensions – and skills to master the field, etc. Such psychological dimensions include personality traits as a key notion (Fernández Fernández & Peralta López, 1998; González Romo, Tejada Tayabas, Martínez Morales, Figueroa Rodríguez, & Pérez Jácome, 2007).

The study of personality in creative individuals has been carried out using two main theoretical approaches. The classical one focuses on the description of fixed traits – personality styles or pathological dimensions. A new approach, based on the paradigm of complexity, has also been posed. It describes the phenomenon by means of a dynamic coexistence of antagonistic tendencies.

### Creative Personality: The Classical Approach

Classical research on personality dimensions in creative persons emphasises the description of certain accentuated and relatively stable traits in such individuals. The methodology employed comprises case studies as well as psychometric developments.

Sometimes the concept of creativity has been held as linked to pathological traits as suggested by Eysenck (1995), especially to a high degree of psychoticism – aspect shared with psychotic patients. Psychoticism would allow the generation of a considerable amount of ideas and associations in short periods of time, effected by the cognitive mechanism of over-inclusion, due to the lack of a cognitive inhibition. This hypothesis has been reinforced by studies reporting high levels of psychoticism in most creative individuals (e.g. Abraham, Windmann, Daum, & Güntürkün, 2005; Burch, Hemsley, Pavelis, & Corr, 2006; Joy, 2008).

Nevertheless, most researchers are interested in the analysis of non-pathological traits related to creativity. From the traditional perspective, the creative profile described by Guilford (1950) and Lowenfeld (1947) is composed of qualities such as sensitivity, receptivity, adaptability to new events, originality, ability to modify the function of an object, synthesis capacity, and an adequate expressivity to transmit much information using very few means. Amabile (1983) highlights self-discipline, ability to postpone satisfaction, perseverance, tolerance towards frustration, independence, a sustained effort and a permanent self-education – either explicit or implicit – by experimentation or by trial and error.

From the same theoretical approach but assuming a more current perspective, Davis (1989) states that creative persons are distinguished by being aware of such attribute in themselves. They are independent and energetic, perceiving in a refined way. They also take risks, are curious, have a good mood, an open mind and artistic sense, appreciate loneliness and they feel attracted to novelty and complexity. Rogers (1975) points out their self-confidence, their openness to experience, nonconformity and occasionally, their cultural mismatch. Gardner (1997) underlines their high motivation and their expressive ability to employ symbolic means, as well as their adequate tolerance towards uncertainty, along with a strong persistence. Other remarkable notes include ignoring fears and a high tendency to socialise.

Recently the concept of creative self-beliefs has been used to explain creative performance (Glăveanu & Tanggaard, 2014). The development of self-beliefs depends on the individual ability to get profit from his/her life events. Such events, as well as the way each person experiences them are determined by some personality traits – extroversion, openness to experience, curiosity, etc. – which have been pointed out as related to creative processes (Karwowski, 2012; Kaufman & Beghetto, 2009; Silvia, Beaty, Nusbaum, Eddington, Levin-Aspenson, & Kwapil, 2014).

#### Creative Personality in College Students

Creative profiles have been studied in college students taken as a whole population, considering pathological and adaptive traits. Besides, differences by major were also analysed in order to comprehend specific features in every group, predicting possible outcomes on achievement.

As hypothesized, psychoticism, hypomania and impulsivity were found as enabling thinking fluency, being a capital feature to produce novel ideas in undergraduates as a whole (e.g. Acar & Runco, 2012; Carlsson, 2002; Fulford, Feldman, Tabak, McGillicuddy, & Johnson, 2013; Sapranaviciute, Perminas, & Sinkariova, 2010; Schuldberg, 2001).

Regarding adaptive traits, a comparative meta-analytical study inventoried a group of traits which were present in the most creative persons when compared to those less creative individuals – autonomy, introversion, openness to new experiences, norm-doubt, self-confidence, self-acceptance, self-driving, ambitiousness, dominance, hostility, and impulsivity (Feist, 1998). Various of these traits, as well as some others were identified in creative college students – independence, extraversion, cognitive control, the willingness to overcome obstacles, tolerance to ambiguity, self-efficacy, and self-discipline (e.g. de Acedo Baquedano & de Acedo Lizarraga, 2012; Dollinger, Palaskonis, & Pearson, 2004; Esfahani, Ghafari, Emami, & Baboli, 2012; Runco & McGarva, 2013; Silvia, Nusbaum, Berg, Martin, & O’Connor, 2009; Sternberg, 2006).

Particularly in fields such as Art, Engineering, Social Sciences, and Physical Education, creativity can be described by extraversion and meticulous attention attributes (Sapranaviciute, Perminas, & Sinkariova, 2010; Zare, Heris, & Bayat, 2011). Students of Business integrate extraversion and openness to experience (Sung & Choi, 2009). Regarding Art students, openness to experience was significantly associated to the ability of acquiring artistic knowledge, creative accomplishments, and daily creative actions. Additionally, these individuals were aware on such traits in themselves (Silvia & Nusbaum, 2012).

Diverse studies have stressed the importance of some creative traits in learning processes in higher education. On one hand, extraversion and openness to experience arose as salient in creative individuals. Openness to experience and extraversion were analysed simultaneously finding a joint effect on the commitment factor composing academic motivation (Komarraju & Karau, 2005). Both stand out as having a direct effect on critical thinking (Clifford, Boufal, & Kurtz, 2004). On the other hand, extraversion was strongly associated to active coping, favouring problem solving (Contreras Torres, Espinosa Méndez, & Esguerra Pérez, 2009).

Researchers have established relationships between certain traits of creative personality and academic achievement in college students in general, as well as by major. Beginning with higher education in general, a meta-analysis reported a positive association between academic achievement and agreeableness, conscientiousness, and openness (Poropat, 2009). Besides, a longitudinal study found a positive effect of conscientiousness on academic achievement (Chamorro-Premuzic & Furnham, 2003).

Additionally, some studies reported the positive influence of several traits observed in creative students on academic achievement – flexibility and openness to experience, efficacy, independence, cognitive control, integrity, honesty, intuition, acceptance of authority, self-confidence, curiosity, concern about other people and disciplined imagination (e.g. Campos & González, 1993; de Acedo Baquedano & de Acedo Lizarraga, 2012; Dollinger, Palaskonis, & Pearson, 2004; Runco & McGarva, 2013; Silvia, Nusbaum, Berg, Martin, & O’Connor, 2009; Sternberg, 2006).

Analyses by major revealed that openness to experience and intuition have been recognised as related to achievement in students of Economics (Chowdhury, 2006; Farsides & Woodfield, 2003). Conscientiousness was positively related to academic achievement in Psychology students as well as in undergraduates of Human Medicine (Ferguson, James, O’Hehir, & Sanders, 2003; Peeters & Lievens, 2005).

The above mentioned have accounted for relevant features describing some personality traits – perceptive, cognitive, attitudinal, motivational and related to interests – hypothetically linked to creativity in higher education. Noteworthy to mention, every result corresponds to the traditional approach of creative personality. The complex perspective proposes a different scenario, entailing two lines. The first one poses a specific configuration of traits – personality, ability, interests – for every academic domain, enhancing learning (Ackerman, 1996; Ackerman, Chamorro-Premuzic, & Furnham, 2011). The second refers to the coexistence of antagonistic personality traits in the same individual. That will trigger opposite behaviours according to the situation (Csikszentmihalyi, 1996), alluding to personality features which particularly distinguish creative people. This study addresses the latter.

### Creative Personality: The Complex Perspective and the Concept of Paradoxical Personality

As stated before, the classical study of creative personality aims at analysing different traits, trying to explain creative behaviours in such individuals. Despite the consensus that there are certain features common to creative people, identifying a homogeneous and unique group of traits seems nearly impossible (Helson, 1996). Still, some regularity was observed in a series of case studies. It was the ability to modify the own actions by the adoption of antagonistic attitudes in situations perceived as completely different.

The hypothesis asserts that human beings initially possess multiple contradictory qualities which become fixed or atrophied by the action of developmental processes. Once atrophied, only one pole of that dialectical will be expressed, configuring a particular personality style. Creative people, instead, will retain both poles, being able to express a wide range of attributes under diverse circumstances, resulting in the notion of *paradoxical personality* (Csikszentmihalyi, 1996). Paradoxical personality involves a whole range of personality styles, which are available to be used in different contexts. Hence static traits lose prominence and behaviours fluctuate around a multiplicity of polarized attributes.

The paradigm of complexity replaces the idea of a creative configuration in adults, featured by invariable notes, by the notion of coexistence of paradoxical tendencies and thoughts. A large majority of people do not experience this coexistence, leading to the hypothesis of a direct relationship between the complexity of personality – represented by the number of paradoxical traits – and the degree of creativity. Thereby, this approach sets aside the notion of creative profile proposing the study of the level of complexity in personality (Haller & Courvoisier, 2010).

The rationale which allowed introducing the concept of paradoxical personality can be situated in previous studies following a case study methodology. Maslow (1973), from the humanistic approach, stated that, by a mechanism of resolution of dichotomies, creative people can sustain two opposite traits in a new synthesis. He highlighted pairs such as altruism-hedonism, instinct-reason, duty-pleasure, and work-play, among others. As this reasoning is applied to every area of human life, it has an impact on every specialisation field as well. For instance, the artist is able to combine opposite colours, shapes or notes which seem incompatible for most people.

McMullan (1976) addressed the conflict between attributes, posing eight polarities – delay closure, convergence/divergence, mindless perception/constructive discontent, detached involvement, disinterested selfishness, confident humility and relaxed attention. More recently, Csikszentmihalyi (1996) joined these ideas under the name of paradoxical personality. He asserted that, though a great variety of qualities might be observed, ten pairs of traits apparently antithetical stand out from the rest in the cases analysed. Although the author did not label these pairs, they could be named as Hyperactivity-Hypoactivity (energetic control), Rationality-Intuition, Responsibility-Irresponsibility, Imagination-Reality, Extraversion-Introversion, Humility-Pride, Masculinity-Femininity (Androgynia), Conservatism-Iconoclasm, Objectivity-Subjectivity (Passion), and Suffering-Pleasure. Not every dichotomy is necessarily present in every creative person. Moreover, the possibility of other new dichotomies not considered in this study stays open.

The notion of paradoxical personality introduces a new way of understanding personality where the relevant issue is the number of contradictory traits present in every individual. The more opposite features present in the same person, the more complex his/her personality will be, and therefore, his/her creativity will be more developed. As mentioned before, the incipient research on the subject is based mainly on case studies, currently showing scarce psychometric developments.

The present study addresses the notion of paradoxical personality, attempting to assess the degree of complexity in which it manifests in college students. Its goals aim at: a) describing paradoxical personality traits in each major, b) analysing possible statistically significant differences in each trait according to majors, c) examining associations between traits and academic achievement as they apply to each specialisation field.

Results will contribute to increase the understanding of local college students, generating new information useful to adapt and to design teaching programs, especially for low achievers, in accordance with their personality features. Besides, it could be employed to guide the students’ decisions according to these personality notes, aiming at the improvement of academic achievement and, in a wide sense, at learning enhancement. That will favour quality and equity in education (Garbanzo Vargas, 2007; Tejedor Tejedor & García Valcárcel, 2007).

## Method

### Design

A descriptive-inferential design was carried out, analysing differences between groups and correlating the behaviour of academic achievement by means of paradoxical traits. Data were gathered using a non-probabilistic sampling (Miles & Banyard, 2007; Scheaffer, Mendenhall, & Lyman Ott, 2007).

### Instruments

#### Personal and Academic Survey

It included information about age, gender, university, faculty, academic major, time elapsed from the beginning of studies, and number of passed classes.

Academic achievement was estimated through the operative definition describing it as academic success (Tejedor Tejedor, 2003). It comprises passing a class, course, term, cycle, or graduation, in the expected time by every institution. That allows the calculation of achievement employing the ratio between the number of passed classes and the time elapsed from application/admission, obtaining as a result an index positively correlated to academic achievement (De Miguel & Arias, 1999). Notwithstanding this measure is open to debate, it has been used following diverse previous studies (e.g. Di Gresia, Porto, Ripani, & Sosa Escudero, 2003; Ibarra & Michalus, 2010; Porto & Di Gresia, 2001).

#### Paradoxical Personality Test

A scale especially developed to assess this concept in college students from Buenos Aires (Freiberg Hoffmann, de la Iglesia, Stover, & Fernández Liporace, 2014) was employed. This measure accounts satisfactory content, face and construct validity evidences, as well as internal consistency and test-retest studies. Following Csikszentmihalyi (1996), it was initially designed to assess 10 paradoxical personality dimensions, but after being analysed by means of principal components in studies conducted with local college students, only 6 paradoxical dimensions were confirmed. The procedure involves a short examination, in which the 6 remaining dimensions of paradoxical personality are measured (Table 1). They are represented by 30 items. Every item consists of an assertion integrating two contradictory traits simultaneously, in order to represent each paradoxical trait in the best possible way (Table A1).

**Table 1:** Paradoxical Personality Dimensions (Csikszentmihalyi, 1996). Description

Dimension	Description
Hyperactivity-Hypoactivity	Persons who are sometimes very energetic and some other times, they remain quiet and silent. They can work continuously for huge periods, and after that they use to take long breaks. They think that a given activity rhythm must be followed by another of reflection and leisure.
Imagination-Reality	These people possess fantastic ideas with a strong anchorage in reality. Their mental models account, at least, a minimal connection to some aspect of reality.
Extraversion-Introversion	These persons enjoy being in a crowd, though sometimes they remain aside and avoid participation. They use to watch or to listen to other persons’ ideas, exchanging impressions with others as well. However they tolerate loneliness, as necessary in the creative process.
Humility-Pride	The most capable persons use to employ self-criticism, sometimes being shy. Some other times they behave arrogantly and contemptuously. This polarity also manifests as ambition-cooperation or disinterest-competitiveness. These individuals use to be ambitious and aggressive but on the other hand they are inclined to subordinate their own projects and interests to benefit the group or the team.
Conservatism-Iconoclasm	This paradox is also represented by rebellion and traditionalism. Creative products require some knowledge and acceptance of values, manners and cultural products which will be transgressed. Rebellion and traditionalism are necessary for allowing the creative process taking place. Being only conservative leaves the field unmodified; and taking chances permanently without referring to the past rarely conduces to a novel production accepted as an improved product.
Suffering-Pleasure	Openness and sensibility expose creative persons to suffering and pain, but to also to pleasure. Inventors possess a low pain threshold. Because of that when an artist has dedicated years to some composition – or a scientist to some development – it is devastating when nobody shows interest. On the other hand, the pleasure they experience when they can work in complete freedom is huge.

Worthy to mention that this format violates two of the main principles proposed in items composition standards (Likert, 1932; Moreno, Martínez, & Muñiz, 2004; Thurstone, 1928). The first one poses to eliminate items containing two opposite propositions, in order to minimize their ambiguity. The second stresses the convenience of designing short sentences to avoid fatigue in examinees. The intentional noncompliance of both rules prioritizes the idea of shaping representative indicators of the kind of behaviour to be assessed (Aiken, 2003; Martínez Arias, 2005). Thus, in order to examine paradoxical behaviours, statements must refer to both opposite poles at once. It is supposed that such type of items will be suitable for persons possessing these paradoxical features, since the content reflects their self-perceptions about their own behaviours. Oppositely, individuals possessing such paradoxical traits in a low degree will experience discomfort towards the content, responding in consequence.

Furthermore, instructions stress that examinees must respond to the whole proposition, and not only to one of its parts. High scores in some dimension would express more flexibility to move from one pole to the other. Lower scores could be associated to more rigid behaviours. It is important to highlight that the scale does not measure every trait separately, but it assesses the alternation of antagonistic behaviours.

Responses use a 5-point Likert scale where 1 represents the less possible agreement with the propositions and 5, the maximum degree.

### Participants

The sample was composed of 350 college students from Buenos Aires – 33.7% males; 66.3% females – with ages ranging from 20 to 35 – *M*_Age_ = 24.28; *SD* = 2.92. This age range represents the 80% of college population according to official statistics (Universidad de Buenos Aires, 2011). Examinees had accomplished half of their studies for their degree programs. The requirement of being in at least the third year of their studies was established as an inclusion criterion. The sample included homogeneous numbers of students (*n* = 50) from 7 different majors of public institutions (Biology, Computer Science, Engineering, Law, Nutrition, Psychology, History of Art).

### Procedures

Data gathering was developed during classes by a trained psychologist. Participants volunteered, not receiving any economical retribution and signing an informed consent. Confidentiality of results and anonymity were always guaranteed, and examinees were informed about the possibility of stopping responses whenever they desired. The study was supported by institutional endorsements.

### Data Analysis

Data were analysed using SPSS 21 package (IBM Corporation, 2012). Since data verified the hypotheses of normality and homoscedasticity in every case, parametric tests were calculated. Besides, and adequate statistical power of the analyses was ensured by estimating the effect size, considering the cut-off values established by Cohen (1977).

## Results

In order to identify paradoxical personality traits in college students from different majors, raw scores were classified according to 5 percentile ranks established for every trait, considering the whole sample. These ranks express the degree in which each trait manifests in every student, given the following positions: low (P_L_), medium low (P_ML_), medium (P_M_), medium high (P_MH_), high (P_H_) (Table 2).

**Table 2:** Paradoxical Personality Traits. Presence According to Raw Scores

Paradoxical Traits	Row scores				
	P_L_	P_ML_	P_M_	P_MH_	P_H_
Hyperactivity-Hypoactivity	18 ≤	19-22	23-25	26-27	≥ 28
Imagination-Reality	6 ≤	7-8	9-10	11-12	≥ 13
Extraversion-Introversion	7 ≤	8-9	10-11	12-14	≥ 15
Humility-Pride	5 ≤	6-7	8	9-11	≥ 12
Conservatism-Iconoclasm	10 ≤	11-13	14-15	16-18	≥ 19
Suffering-Pleasure	13 ≤	14-17	18-21	22-26	≥ 27

*Note.* P*i* = *i*-th percentile; P_L_ = P1-P19; P_ML_ = P20-P39; P_M_ = P40-P59; P_MH_ = P60-79; P_H_ = P80-P100.

Next, means and standard deviations for every trait were calculated by major, using raw scores as well. After that, they were classified according to the former categories (Table 2).

Generally speaking each trait manifested in every major within the range of medium values, except for cases such as the Hyperactivity-Hypoactivity pair, which appeared showing a medium low level in Psychology students. The trait Humility-Pride manifested in Computer Science and Industrial Engineering students in a medium high degree, and in Nutrition students as medium low. The pair Conservatism-Iconoclasm arose in a medium high level in Nutrition students, and medium low in History of Art students. Finally Computer Science students showed the trait Suffering-Pleasure with a medium low presence whereas History of Art students presented it in medium high way (Table 3).

**Table 3:** Paradoxical Personality Traits by Major

Career	Statistic	Paradoxical Personality Trait					
		HH	IR	EI	HP	CI	SP
Biology	*M*	25.16	10.16	10.72	8.12	14.04	20.44
	*SD*	4.39	3.31	3.54	3.03	4.07	6.91
	Presence	P_M_	P_M_	P_M_	P_M_	P_M_	P_M_
Computer Science	*M*	24.02	10.36	11.64	9.40	14.50	17.72
	*SD*	5.08	3.27	3.73	2.32	3.77	6.00
	Presence	P_M_	P_M_	P_M_	P_MH_	P_M_	P_ML_
Engineering	*M*	24.26	10.54	11.26	9.78	15.56	19.00
	*SD*	5.33	3.11	4.26	2.97	3.23	6.93
	Presence	P_M_	P_M_	P_M_	P_MH_	P_M_	P_M_
Law	*M*	24.24	9.68	10.90	8.86	15.74	21.66
	*SD*	4.73	3.37	3.77	2.93	3.57	5.44
	Presence	P_M_	P_M_	P_M_	P_M_	P_M_	P_M_
Nutrition	*M*	23.08	9.38	11.18	7.42	16.20	20.76
	*SD*	5.34	3.63	3.97	2.90	3.98	7.16
	Presence	P_M_	P_M_	P_M_	P_ML_	P_MH_	P_M_
Psychology	*M*	22.95	9.62	10.60	8.70	14.82	18.50
	*SD*	5.94	3.68	4.27	3.24	4.57	5.94
	Presence	P_ML_	P_M_	P_M_	P_M_	P_M_	P_M_
History of Art	*M*	24.20	10.68	11.04	8.74	13.66	22.80
	*SD*	5.40	2.82	3.59	3.03	4.39	6.10
	Presence	P_M_	P_M_	P_M_	P_M_	P_ML_	P_MH_

*Note.* HH = Hyperactivity/Hypoactivity; SP = Suffering/Pleasure; CI = Conservative/Iconoclast; EI = Extraversion/Introversion; IR = Imagination/Reality; HO = Humility/Pride; P*i* = *i*-th percentile; P_L_ = P1-P19; P_ML_ = P20-P39; P_M_ = P40-P59; P_MH_ = P60-79; P_H_ = P80-P100.

Analysing differences in paradoxical traits by major, significant results were found for Suffering-Pleasure (SP), favoring History of Art students when compared to students of Engineering, Psychology and Computer Science, and between the last group compared to Law students, all of them with moderate effect sizes (ES) (*η^2^* = .066). The pair Conservatism-Iconoclasm (CI) also showed differences. They were exhibited between the Nutrition and History of Art groups, favoring the first, with a low-moderate ES (*η^2^* = .046). The last difference verified corresponded to Humility-Pride (HP) distinguishing students of Nutrition versus Engineering and Computer Science, favoring these two last groups, with a moderate ES (*η^2^* = .058) (Table 4).

**Table 4:** Paradoxical Personality Traits. Differences by Major.

Career	Statistic	Paradoxical Personality Traits					
		HH	SP	CI	EI	IR	HP
**Biology** (*n* = 50)	*M*	25.16	20.44^abc^	14.04^ab^	10.72	10.16	8.12^ab^
	*SD*	4.39	6.92	4.07	3.54	3.31	3.03
**Computer Science** (*n* = 50)	*M*	24.02	17.72^a^	14.50^ab^	11.64	10.36	9.40^b^
	*SD*	5.08	6	3.77	3.73	3.27	2.32
**Engineering** (*n* = 50)	*M*	24.26	19^ab^	15.56^ab^	11.26	10.54	9.78^b^
	*SD*	5.34	6.94	3.24	4.26	3.11	2.97
**Law** (*n* = 50)	*M*	24.26	21.66^bc^	15.74^ab^	10.90	9.68	8.86^ab^
	*SD*	4.73	5.44	3.58	3.77	3.37	2.93
**Nutrition** (*n* = 50)	*M*	23.08	20.76^abc^	16.20^b^	11.18	9.38	7.42^a^
	*SD*	5.35	7.16	3.98	3.98	3.63	2.90
**Psychology** (*n* = 50)	*M*	22.96	18.50^ab^	14.82^ab^	10.60	9.62	8.70^ab^
	*SD*	5.95	5.95	4.58	4.28	3.68	3.24
**History of Art** (*n* = 50)	*M*	24.20	22.80^c^	13.66^a^	11.04	10.68	8.74^ab^
	*SD*	5.40	6.10	4.40	3.59	2.82	3.03
	Levene’s Sig.	.128	.189	.123	.549	.509	.068
	*F*	1.055	4.025	2.786	.409	1.140	3.543
	*p*	.389	**.001**	**.012**	.873	.338	**.002**
	*η^2^*	.018	.066	.046	.007	.020	.058

*Note.* Different letters indicate statistically significant differences (*p* < .05) between groups.

Going on with the analysis of the relationship between paradoxical personality traits and academic achievement, the latter was calculated as a metric variable by means of the ratio between the number of passed classes and the number of years elapsed from the admission (De Miguel & Arias, 1999). After that each score representing each bipolar trait of paradoxical personality was correlated to academic achievement for each major (Table 5).

**Table 5:** Relationship Between Paradoxical Personality Traits and Academic Achievement for Majors

Career	Paradoxical Personality Traits					
	HH	SP	CI	EI	IR	HP
**Computer Science** (*n* = 50)		-.417**				
**Nutrition** (*n* = 50)	-.290**					
**Psychology** (*n* = 50)			.327*		.318*	.295*

Correlations verified significant results for paradoxical personality traits and academic achievement in the following majors: Computer Sciences, Nutrition and Psychology. Additionally, Computer Sciences and Nutrition showed negative associations between academic achievement and paradoxical traits such as Suffering-Pleasure for Computer Sciences and Hyperactivity-Hypoactivity for Nutrition. On the contrary, Psychology obtained positive associations between achievement and the pairs Conservatism-Iconoclasm, Imagination-Reality and Humility-Pride.

## Discussion

The study analysed paradoxical personality traits in college students from different academic majors. The ultimate goal aimed at the description of such traits, in order to establish if the idea of paradoxical personality – hypothesised as related to creative skills – could be useful when academic achievement matters. Creative skills are posed as valuable by academic institutions considering educational outcomes in future professional life, where graduates must deal with unique problems permanently. Following Crozier (2001), academic achievement is influenced by the interaction between personality traits and teaching methods. Hence, understanding these traits, and mainly those possibly linked to creativity, will contribute to adapt teaching to individual features, enhancing the final result of the whole learning process, as well as its future transference to professional situations.

First, the presence of each bipolar trait was described in each major. Generally speaking each attribute manifested in a medium degree in students of every discipline. A few exceptions were found though always moving within the medium range. Most of them showed in a medium degree, whereas a few bipolarities manifested in a medium-low or medium-high degree. There were no antithetical pairs exhibiting huge gaps concerning the degrees on which they appeared in any major. Regarding this, significant differences between majors were analysed in order to identify distinctive features in students of each field, which could be employed as a guide to conduct teaching methods. When teaching strategies match students personality traits, academic achievement will improve, since learning processes could be enhanced (Crozier, 2001).

As for results obtained by the analyses of differences by major, History of Art students distinguished significantly in the Suffering-Pleasure dimension, overcoming Engineering and Psychology, and surpassing even more Computer Science students. This implies that History of Art students will be open and sensible, tending to experience pain as well as pleasure easily. This configuration facilitates creative activities, but teachers must be aware of the impression that their actions may cause in this group. Because of that when a student has dedicated much effort to any particular activity it could be demoralising if nobody shows interest or when reprobation arrives. Freedom and positive rewards seem to be the best strategy.

Another difference verified by this analysis consisted in higher scores in Conservatism-Iconoclasm for Nutrition students when compared to History of Art. This finding could be beneficial for Nutrition classes since, on one hand, Conservatism leads to the acceptance of values, manners and cultural products, which is important when comprehending food habits in clients. Iconoclasm, on the other hand, may favour healthier modifications in nourishment plans, even within a particular cultural framework.

Significant differences were verified for Humility-Pride, favouring the Engineering and Computer Science group compared to the Nutrition one. Individuals showing this pair use to be ambitious and aggressive being, in addition, capable to subordinate their own interests to the group. This way, Engineering and Computer Science students manifest these two skills, which can be exploited and stimulated in class. Teamwork as well as individual activities arise as habitual ways of pursuing goals in these professional fields. Engineering and Computer Science professional profiles proposed by academic institutions allude to collaborative activities, interacting with diverse organisational sectors. Conversely, profiles for Nutrition prioritize bonding with clients or patients (Facultad de Ciencias Exactas y Naturales, 2014; Facultad de Ingeniería, 2014; Facultad de Medicina, 2014).

Concerning the correlational analysis of paradoxical personality traits and academic achievement, statistically significant associations were found for three of the seven majors analysed, Computer Science, Nutrition, and Psychology.

Regarding Computer Science students, academic achievement decreased when the Suffering-Pleasure dimension manifested in a higher degree. The description of personality traits by major and the exam of group differences have shown less presence of the Suffering-Pleasure bipolarity in Computer Science students when compared to the rest of the sample. The first analysis found a medium-low presence of the trait (Table 3), whereas the second reported that this group was surpassed by the History of Arts and Law groups in such pair (Table 4). This Suffering-Pleasure bipolarity is posed to be triggered by a higher openness and sensitivity, sometimes considered as a pathological state configuring an emotional disorder. Even existing some evidence on the link between emotional instability and creative skills (de Acedo Baquedano & de Acedo Lizarraga, 2012), in this case the former could obstruct academic achievement, since it is associated to isolation and feelings of incomprehension, which might affect persistence. Computer Science teachers should be trained in identifying these features in students, in order to suggest some consultations with tutors or counsellors. Such guidance might help unstable students in managing their emotions, thereby coping successfully with academic stress. Furthermore, teachers might avoid excessive criticism, providing more subtle feedbacks. They must encourage students to let them gaining stability, motivation and perseverance, along with tolerance towards frustration.

Correlations calculated for the group of Nutrition indicated that academic achievement decreased when the Hyperactivity-Hypoactivity bipolarity increased. This might be expressing that these students have a little control of their own energies, which seem to be regulated by external factors (e.g., academic deadlines). It appears as logical when one of the aspects valued in the major profile alludes to goals accomplishment and deadlines. Teachers must structure classes as much as possible, presenting examination dates in advance, as well as paths and goals to be accomplished in class. Extraordinary activities and a permanent monitoring of learning could be added. This would allow students to get a progressive self-management of time, efforts, results, and, finally, of the own learning process. Nutrition students require permanent monitoring, providing study aids and diverse types of practices. B-learning classes come up as profitable, allowing the integration of online exercises and materials, on one hand, and contents included in standard classes, on the other.

The analysis developed for the Psychology group identified several associations indicating that academic achievement increased when Humility-Pride, Conservatism-Iconoclasm and Imagination-Reality rose. Regarding the Humility-Pride pair, Psychology students seem to be ambitious and individualist, being in the disposition to subordinate their personal interests to benefit the group at the same time. In view of that, teachers must promote individual and group activities, pursuing a balance between them. As for the Conservatism-Iconoclasm pair, future psychologists working in any field must deal with traditions, accepted values, beliefs and thoughts, designing guidance able to integrate that to changes, new ideas, novel solutions and different possible scenarios in people’s lives. They must base guidance on recovering the existent, improving it according to current and actual life in a creative way. This applies to clinical situations, but to educational and organisational fields as well, and even referring to public health campaigns. For instance Imagination-Reality arises as a useful bipolarity in view of that Psychology curricula comprise two stages in learning (Ministerio de Educación, Educación Superior, 2009). The first one is aimed at the acquisition of knowledge entailing a wide variety of theoretical models, and the second, at the set in motion of diverse practices in different psychological professional fields. Hence the hypothesis of Imagination-Reality as enabling the transference of theoretical contents to professional situations seems reasonable. Teachers could be trained to integrate such types of contents in despite of the fact that habitually they are presented in separate classes.

Regarding limitations of the study, the first one appears to be the small number of majors assessed. Future developments will expand their diversity in order to represent local college population, as well as to increase the number of participants as a whole. In addition, the measure employed to estimate academic achievement remains, indeed, as an object of debate. It ponders performance according to academic schedule – number of passed classes and time elapsed from admission – setting aside other indicators equally important – grade point average, number of credits, rate of success, delay and dropout, among others (De Miguel & Arias, 1999; Martín, García, Torbay, & Rodríguez, 2008). Further investigations must add some of these indicators in order to estimate academic achievement, therefore reaching a higher level of accuracy.

Limitations referred to the concept of paradoxical personality also arise as matter of concern. First, as a result of previous factor analyses the study examined only six of ten paradoxical pairs originally assessed for the first version of the scale (Freiberg Hoffmann, de la Iglesia, Stover, & Fernández Liporace, 2014), excluding as well other antagonistic traits potentially significant for college students. Developments identifying new possible dichotomies must be carried out furtherly. Additionally there are limitations linked to the scarcity of literature on paradoxical personality studies in college students. That fact has determined that the interpretation of the results obtained were performed regarding intrinsic features of majors and the professional profiles expected in the syllabi in force. However, this lack of literature reinforces the contribution of this study, since it becomes an antecedent in the field, tabling this research line as a matter of interest. About dimensions posed by researchers (López, Corbalán, & Martínez, 2006; MacKinnon, 1978; Pascale, 2005) when analysing creative dimensions – person, product, process, environment – this study has analysed only the person aspect. Examining the rest of them will contribute to gather new evidence to test models on creativity in college students.

Besides, these results provide valuable practical information for professionals as well. Paraphrasing Csikszentmihalyi (1996), individuals can be identified as more or less – or diversely – creative taking into account the number – and quality – of antagonistic features exhibited, thus distinguishing configurations, since not every opposite pair manifests in the same person. Considering this, the study identified bipolar traits of paradoxical personality useful to characterise students from diverse fields or specialisation areas, paying close attention to those related to academic achievement.

Findings show that not every creative bipolar trait is related positively to academic achievement. Moreover, some of them could be detrimental. This is the point where the debate about creativity in the academic milieu comes up. Even when creativity is posed as a valuable and desirable feature in learning as well as in professional profiles, academic institutions do not encourage creative skills in students. Hence, some questions emerge: Are teachers trained to teach to be creative being creative at the same time? Is it possible to verify the hypothesised relationship between paradoxical personality and creative skills? Is it possible to train creative skills based on paradoxical personality traits? Creative skills will increase if students achieve more flexibility to fluctuate from one paradoxical pole to the other? Anyhow, describing these features in our students, identifying differences between groups and establishing how these personality traits may relate to academic achievement appear as a preliminary step in analysing this notion. Complexity involved in paradoxical traits seems logical when creative skills are regarded. These findings must be useful as a first stage, but they must be investigated in depth. As a beginning, the information presented might be useful as a practical resource in class. If academic institutions pay attention to individual differences in personality dimensions, teaching strategies could be adapted to particular cases or specific groups, enhancing educational efficacy. It is expected that what was reported here will help educational psychologists, pedagogues and teachers to make decisions oriented to improve academic achievement, reducing dropout rates and academic failure.

This research line is expected to be extended in order to delve deeper into this matter in college populations, always taking into account further applications in academic and labour scenarios.

## Appendix

**Table A1:** Paradoxical Personality Test. Preliminary Translation of Items Into English (not Being Tested or Validated)

Items in Spanish	Items in English
1. Soy una persona que ante la menor cosa negativa sufro, y ante la menor cosa positiva me alegro.	1. Facing the minimal negative event, I suffer, and facing the minimal positive event, I feel happy.
2. Creo ser una persona muy sensible y susceptible a experimentar placer y dolor.	2. I think I am a very sensitive and susceptible person, when experiencing pleasure and pain.
3. Cuando realizo mis actividades, muchas veces me invade una gran satisfacción y olvido mis preocupaciones, pero en ocasiones el sufrimiento se acrecienta.	3. When I develop my activities, I often feel a huge satisfaction and I leave my worries away, but occasionally suffering begins to increase.
4. Tengo momentos de gran melancolía que suelen saltar hacia momentos de gran felicidad.	4. I have moments of melancholy which sometimes change towards moments of great joy.
5. Cuando las cosas no me salen suelo sufrir bastante y permanecer inmóvil, pero a veces, por el contrario, tomo mayor energía y lo afronto.	5. When I cannot do things well, I use to suffer quite a bit and to freeze, but sometimes, on the contrary, I take hold and I cope with those things.
6. Soy muy susceptible a experimentar placer cuando se me elogia, y gran sufrimiento cuando se me critica.	6. I am so inclined to experience pleasure when somebody praises me, and a huge suffering when somebody criticises me.
7. A menudo experimento el sufrimiento y el placer con gran facilidad.	7. I often experience suffering and pleasure very easily.
8. Por momentos hago muchas cosas a la vez, y por momentos no hago nada.	8. Sometimes I do a lot of things at once, and sometimes I do nothing.
9. Soy una persona que puede pasar largas horas en actividad, pero que a la vez le gusta dormir otra buena cantidad de tiempo.	9. I am a person who can spend long time in activity, but who likes to sleep similar periods of time.
10. Suelo dedicar gran cantidad de energía a mis actividades durante períodos de tiempo extensos, y posteriormente tomo prolongados descansos.	10. I use to spend a big amount of energy in my activities for long periods, and later I take long periods to rest.
11. Tengo por costumbre dedicar tiempo al ocio luego de haber trabajado mucho.	11. I use to spend time in leisure after long periods working.
12. Dedico grandes períodos de tiempo a la reflexión y al ocio, para luego poder trabajar arduamente por grandes lapsos de tiempo.	12. I spend long periods to reflexion and leisure, to could work hard for long periods after that.
13. Puedo permanecer atento y concentrado durante mucho tiempo, y disperso y desconcentrado durante otro.	13. I can stay concentrated and focused during long periods, and dispersed and unfocused by similar periods.
14. Creo poder concentrarme cuando lo necesito, sin embargo cada tanto me distraigo con facilidad.	14. I think I can stay concentrated when I need it; however I am distracted from time to time.
15. Generalmente soy bastante tímido, pero por momentos soy bastante atrevido.	15. I am generally quite shy, but sometimes I am quite audacious.
16. En ciertas circunstancias soy una persona muy introvertida, pero en otras soy muy extrovertida.	16. Under some circumstances I am very introverted, but sometimes I am very extroverted.
17. En ciertas reuniones suelo quedarme callado, pero en otras suelo ser muy participativo.	17. In some social situations I use to remain silent, but in some others I use to participate a lot.
18. Estando en reuniones suelo enfrascarme en mí mismo e ignorar a las demás personas, pero a veces soy muy participativo.	18. Being in parties or reunions I use to be introspective, ignoring people, but sometimes y participate a lot.
19. Soy una persona con muchas ideas fantasiosas, pero que no siempre se deja llevar por ellas.	19. I am a person who has many fanciful ideas, but who not always permits being pulled by them.
20. Suelo tener ideas muy fantasiosas con poca aprobación, e ideas muy corrientes con mucha aceptación.	20. I use to have pretty fanciful ideas with very little approval, and so ordinary ideas which are strongly accepted.
21. Por momentos intento crear nuevas realidades, pero otras veces prefiero quedarme cómodo en esta realidad preexistente.	21. Sometimes I try to create new realities, but some other times I prefer to stay comfortably in the actual reality.
22. Tengo producciones novedosas que suelen adaptarse a la realidad, aunque otras veces no se ajustan tanto.	22. I have got novel productions which use to be adapted to reality, though some other times they do not adapt so well.
23. Me siento seguro siendo tradicional, pero sé que a veces es bueno probar cosas diferentes.	23. I feel safe being traditional, but I know that sometimes it is good to try different things.
24. Suelo ser consciente de la importancia del aporte previo de otras personas a mis actividades, aunque otras veces creo que mis logros son solo gracias a mi esfuerzo.	24. I use to be aware of the importance of previous contributions from other people to my activities, although some other times I think that my achievements are only due to me.
25. Suelo explorar nuevas ideas mediante ensayo y error, aunque a veces prefiero hacer las cosas del modo más conocido y estándar posible.	25. I use to explore new ideas by means of trial and error, although sometimes I prefer doing things in the most familiar and standard way as possible.
26. Creo ser una persona bastante tradicional, aunque a veces me gusta hacer cosas fuera de lo común.	26. I think I am a very traditional person, although sometimes I like to do things out of the ordinary.
27. Trato de no alejarme de los convencionalismos por temor al fracaso, pero en ocasiones tomo coraje y emprendo actividades innovadoras.	27. I intend not to get away from conventions because of my fear to failure, but sometimes I take courage and I start with innovative activities.
28. En determinadas ocasiones soy una persona bastante autocrítica, y a veces me comporto un poco arrogante.	28. Occasionally I am quite self-criticism person, and sometimes I behave arrogantly.
29. En ciertas circunstancias me siento superior a los demás, pero en otras me siento demasiado inferior.	29. Sometimes I feel superior to other people, but some other times I feel I am too much inferior.
30. A veces me comporto como una persona muy competitiva pero, en ciertas ocasiones, soy bastante cooperativo.	30. Sometimes I behave as a very competitive person, but occasionally I am quite helpful.
